# Embryonic Stem Cell-Like Subpopulations in Venous Malformation

**DOI:** 10.3389/fmed.2017.00162

**Published:** 2017-10-04

**Authors:** Elysia M. S. Tan, Sam Duro Siljee, Helen D. Brasch, Susana Enriquez, Swee T. Tan, Tinte Itinteang

**Affiliations:** ^1^Gillies McIndoe Research Institute, Wellington, New Zealand; ^2^Centre for the Study and Treatment of Vascular Birthmarks, Wellington Regional Plastic, Maxillofacial and Burns Unit, Hutt Hospital, Wellington, New Zealand

**Keywords:** venous malformation, embryonic, stem cells, markers, pathogenesis

## Abstract

**Background:**

Venous malformation (VM) consists of a network of ectatic anomalous thin-walled venous channels. A role for an activating TIE2 mutation in the development of the dilated luminal vessels in VM, and its proposed involvement of embryonic stem cells (ESCs), led us to investigate the expression of ESC markers in subcutaneous VM (SCVM) and intramuscular VM (IMVM).

**Methods:**

Formalin-fixed paraffin-embedded sections of SCVM from seven patients and IMVM samples from seven patients were analyzed for the expression of Nanog, pSTAT3, OCT4, SOX2, SALL4, and CD44, using 3,3′-diaminobenzidine (DAB) immunohistochemical (IHC) staining. All these samples did not express lymphatic marker D2-40. NanoString mRNA analysis and RT-PCR were performed on snap-frozen samples of SCVM (*n* = 3) and IMVM (*n* = 3) from the respective original cohorts of patients included in DAB IHC staining. To confirm co-expression of two proteins, immunofluorescent (IF) IHC staining on two representative samples of IMVM and SCVM samples from the original cohorts of patients included for DAB IHC staining was performed.

**Results:**

DAB IHC staining demonstrated expression of all of the above ESC markers in both SCVM and IMVM samples. IF IHC staining showed that these markers were localized to the endothelium within these lesions and that Nanog, pSTAT3, SOX2, and CD44 were also expressed by cells outside of the endothelium. NanoString mRNA analysis confirmed transcription activation of pSTAT3, OCT4, and CD44. RT-qPCR confirmed transcription activation of Nanog, SOX2, and SALL4.

**Conclusion:**

Our findings support the presence of two ESC-like subpopulations, one within and one outside of the endothelium, of both SCVM and IMVM. Given that the endothelial ESC-like subpopulation expresses the more primitive marker, OCT4, it is exciting to speculate that they give rise to the non-endothelial subpopulation.

## Introduction

Vascular anomalies are classified by the International Society for the Study of Vascular Anomalies classification system into vascular tumors of which infantile hemangioma is the most common, and vascular malformations of which venous malformation (VM) is the most common (1, 2). Vascular malformations may affect arteries, veins, lymphatics, and capillaries singly or in combinations (1).

Venous malformation, which affects 1% of the population (3), is composed of ectatic anomalous venous channels, lined by flat endothelial cells (ECs) (3–5) with absent or deficient smooth muscle cells (SMCs) within the thin vessel walls (3, 5). VM is present at birth, although it may not be noticed until later in life (3–5). It grows proportionately with the child and may suddenly expand in response to hormonal changes, trauma, or incomplete excision (2–4). VM affects different topographic regions and tissues, commonly involving the subcutaneous tissues and less commonly muscle (6). Subcutaneous VM (SCVM) usually presents as a compressible bluish swelling, whereas intramuscular VM (IMVM) often presents later in life with swelling, pain, or loss of function (5). Approximately 40% of VM occur in the head and neck region, 40% in the extremities, and 20% on the trunk (3, 6).

Management of VM is generally unsatisfactory especially for extensive lesions and includes observation, elastic support (7), low-dose aspirin (7), sclerotherapy, such as ethanol sclerotherapy (8, 9), surgery, or a combination of these treatments (10–12).

The pathogenesis of VMs has not been fully elucidated (2, 13), although recent reports have demonstrated a role for mutations of TIE2-L914F (14) and PIK3CA (15) in the biology of these lesions. VMs are mostly sporadic, with 1.2% being familial (16). A mutation in the TIE-2 gene, a receptor for angiopoietin 1 (Ang-1) expressed almost exclusively on ECs, has been identified in familial (17), and in up to 50% of sporadic (18), VM cases. Activating mutations of this tyrosine kinase receptor results in a ligand-independent hyperphosphorylation (14). Vikkula et al. (19) suggest that the TIE2 mutation in ECs may reduce SMC ligand expression causing a local uncoupling between normal SMC recruitment and the proliferation of ECs.

We have recently demonstrated the expression of components of the renin–angiotensin system (RAS): prorenin receptor (PRR), angiotensin-converting enzyme (ACE), angiotensin II receptor 1 (ATIIR1), and potentially angiotensin II receptor 2 (ATIIR2), in SCVM and IMVM, which suggests a role for the RAS in the biology of VM (20). ATIIR1 is responsible for the proangiogenic effects of ATII, which may contribute to the increased density of abnormal venous channels within VM (20). The presence of ATIIR2 may indicate cellular differentiation determination, as proposed by Zambidis et al. (21). Furthermore, ACE is a marker for primitive human pluripotent stem cell-derived hemangioblasts (20). The expression of ACE on the endothelium of VM (20) and the increased expression of stem cell marker *c*-kit within smaller lesional vessels in blue rubber bleb nevus syndrome (22), a subtype of VM, led us to speculate whether this reflected a primitive phenotype for this endothelium of VM.

A role for TIE2 activation in the formation of increased vessel lumen in vasculature derived from embryonic stem cells (ESCs) (23) parallels the vasculature seen in VMs and led us to hypothesize the expression of a primitive population in endothelium of both SCVM and IMVM.

This study aimed to identify a putative primitive population by their expression of ESC markers, such as Nanog, pSTAT3, OCT4, SOX2, SALL4, and CD44, in SCVM and IMVM.

## Materials and Methods

### Tissue Samples

Previously untreated, SCVM tissue samples from seven patients and IMVM samples from seven patients with a mean age of 22.9 (range, 1.2–54) and 21.1 (range, 8–30) years, respectively, were sourced from the Gillies McIndoe Research Institute Tissue Bank and used in a study approved by the Central Health and Disability Ethics Committee (ref. no. 13/CEN/130). Written informed consent was obtained from the participants.

### Histology and Immunohistochemical (IHC) Staining

Hematoxylin and eosin (H&E) staining was performed on 4-μm thick formalin-fixed paraffin-embedded sections of SCVM (*n* = 7) and IMVM (*n* = 7) samples to confirm the presence of VM tissues on the slides by an anatomical pathologist (Helen D. Brasch). Negative staining for D2-40 (1:100; cat# M3619, Dako, Glostrup, Denmark) was performed in all cases at the Department of Pathology at Hutt Hospital to exclude lymphatic malformation.

3,3′-Diaminobenzidine (DAB) IHC staining for primary antibodies, such as Nanog (1:100: cat# ab80892, Abcam, Cambridge, UK), pSTAT3 (1:100; cat# 9145, Cell Signaling Technology, Danvers, MA, USA), OCT4 (1:30; cat# MRQ-10, Cell Marque, Santa Cruz, CA, USA), SOX2 (1:200, cat# PA1-094, Thermo Fisher Scientific, Waltham, MA, USA), SALL4 (1:30; cat# CM385M-16, Cell Marque, Rocklin, CA, USA), and CD44 (1:1,500; cat# MRQ-13, Cell Marque), was performed on the SCVM and IMVM tissue sections using the Leica Bond Rx auto-stainer (Leica), as previously described (24). Nanog was stained using the ImmPACT NovaRED Peroxidase Substrate Kit (cat# SK-4805, Vector Laboratories, Burlingame, CA, USA) and the ImmPRESS Excel Amplified HRP Polymer Staining Kit (cat# MP-7601, Vector Laboratories). To confirm co-expression of two proteins, immunofluorescent (IF) IHC staining on two representative samples of IMVM and SCVM samples from the original cohorts of patients included for DAB IHC staining was performed with the same primary antibodies at the same concentrations was performed with CD34 (ready-to-use; cat# PA0212, Leica, Newcastle upon Tyne, UK) and ERG (1:200; cat# EP111, Cell Marque), as appropriate endothelial markers. Appropriate secondary antibodies, such as Vectafluor Excel anti-rabbit 594 (ready-to-use; cat# VEDK-1594, Vector Laboratories) and Vectafluor Excel anti-mouse (ready-to-use; cat# VEDK2488, Vector Laboratories) combinations, were used for IF IHC detection. All antibodies were diluted with Bond TM primary antibody diluent (cat# AR9352, Leica). All IHC experiments were performed as single runs.

Positive human controls tissues used were seminoma for Nanog, SALL4, and OCT4; skin for SOX2; and tonsil for pSTAT3 and CD44 (25, 26). To determine the specificity of the primary antibodies, appropriate negative controls consisting of combined Flex Negative Control Mouse (ready-to-use; cat# IR750, Dako, Carpinteria, CA, USA) and Flex Negative Control Rabbit (ready-to-use; cat# IR600, Dako) staining was performed on VM tissues.

### Microscopy

All DAB IHC-stained slides were viewed, and the images were captured using an Olympus BX53 light microscope fitted with an Olympus DP21 digital camera (Tokyo, Japan). IF IHC-stained slides were viewed, and the images were captured using an Olympus FV1200 biological confocal laser-scanning microscope (Tokyo, Japan).

### NanoString mRNA Analysis

NanoString mRNA analysis was performed on snap-frozen samples of SCVM (*n* = 3) and IMVM (*n* = 3) from the respective original cohort of patients included in DAB IHC staining, as previously described (20). Probes for the genes encoding STAT3 (NM_139276.2), OCT4 (NM_002701.4), and CD44 (NM_001001392.1) and the housekeeping gene GAPDH (NM_002046.3) were designed and synthesized by NanoString Technologies (NanoString Technologies, Seattle, WA, USA). NanoString mRNA analysis was performed as a singular run.

NanoString data were analyzed using SPSS (v22, IBM) and validated with nSolver™ software (NanoString Technologies) using standard settings, normalized against the housekeeping gene. Charts were made with Excel.

### RT-qPCR

Total RNA was isolated from formalin-fixed paraffin-embedded samples of SCVM (*n* = 3) and IMVM (*n* = 3) from the original cohorts of patients included for DAB IHC staining, using the RNeasy FFPE Kit (cat# 73504, Qiagen, Hilden, Germany) with DNase digest and the QIAcube system (Qiagen). Total RNA quantity and quality were determined using NanoDrop 2000 (Thermo Fisher Scientific, Waltham, MA, USA). Reverse transcription reactions were performed using the iScript Reverse Transcription Supermix (Bio-Rad, Hercules, CA, USA). The expression of stem cell markers was detected using gene-specific TaqMan (Thermo Fisher) primers probes (SOX2: Hs01053049_s1; SALL4: Hs00360675_m1; Nanog: Hs04399610_g1; GAPDH: 4333764T) with the Rotor-Gene Multiplex RT-PCR Kit (cat# 204974, Qiagen). All measurements were performed in duplicate. Gene expression was determined by the Relative Standard Curve Method, using GAPDH as an endogenous control. Graphs were generated with Microsoft Excel, and results are shown as relative expression.

### Statistical Analysis

The mean levels of mRNA expression for each gene investigated in SCVM vs IMVM were subjected to *t*-test for quality of means using SPSS v 22, to determine and significant differences.

## Results

### Histology and DAB IHC Staining

**Figure 1:**
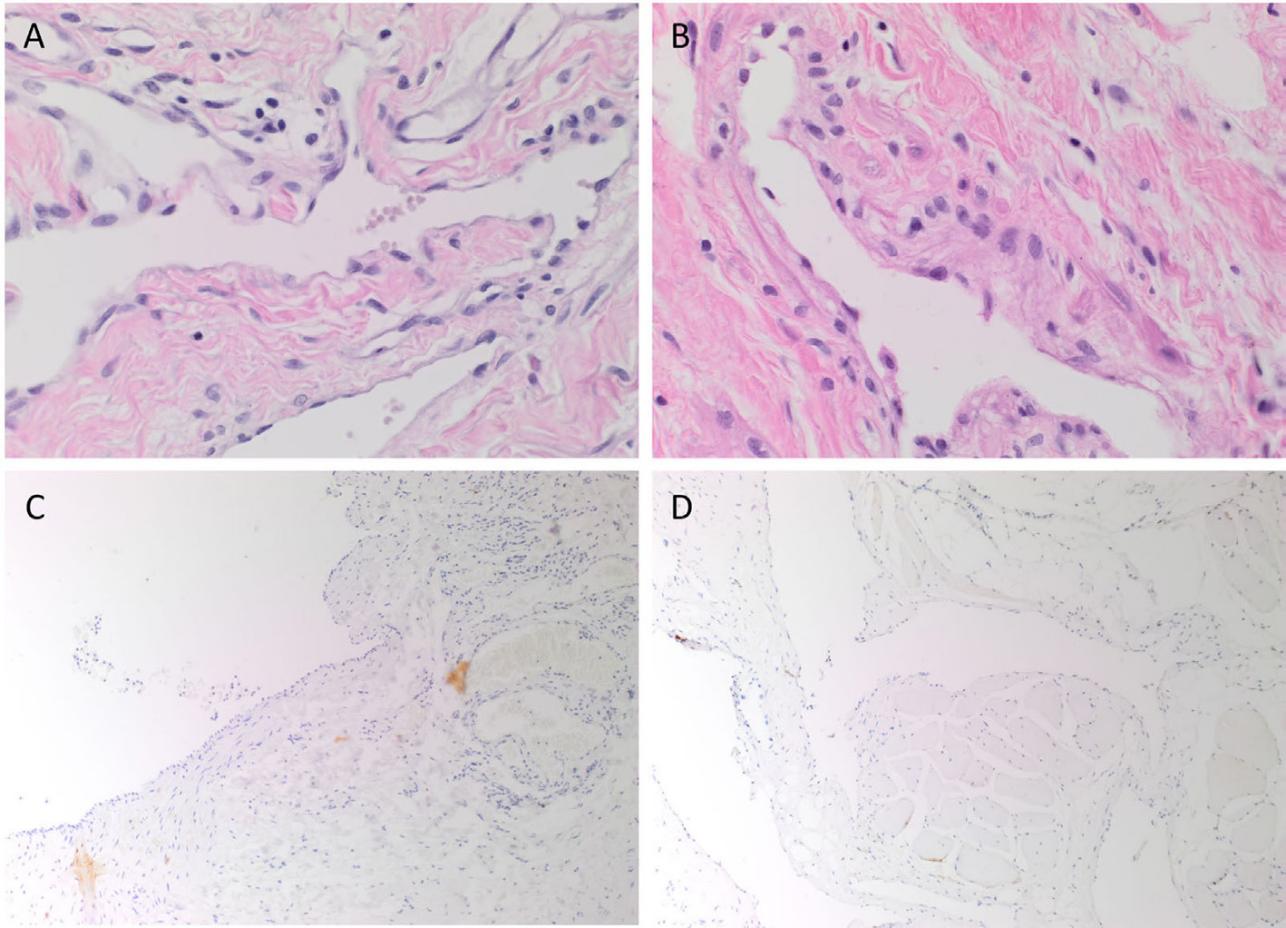
Representative hematoxylin and eosin stained subcutaneous venous malformation (SCVM) **(A)** and intramuscular venous malformation (IMVM) **(B)** sections demonstrating the characteristic ectatic venous channels. Representative sections of SCVM **(C)** and IMVM **(D)** showing minimal staining for D2-40 [**(C,D)**, brown]. Nuclei were counterstained with hematoxylin [**(A–D)**, blue]. Original magnifications: 400× **(A,B)** and 100× **(C,D)**.

Venous malformation tissues, characterized by ectatic venous channels in both SCVM (Figure 1A) and IMVM (Figure 1B), were identified by H&E staining. The SCVM (Figure 1C, brown) and IMVM (Figure 1D, brown) lesions used in this study did not or minimally expressed D2-40, a lymphatic marker.

**Figure 2:**
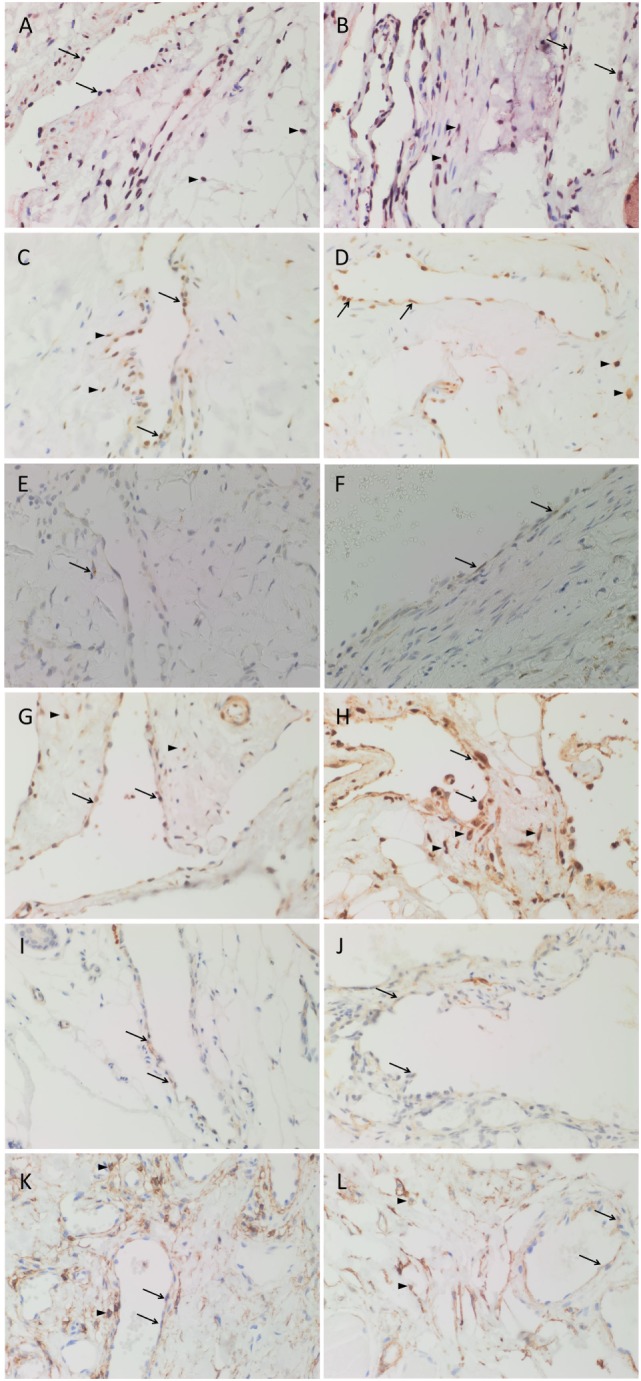
3,3′-Diaminobenzidine immunohistochemical-stained images demonstrating the expression of Nanog [**(A,B)**, red], pSTAT3 [**(C,D)**, brown], OCT4 [**(E,F)**, brown], SOX2 [**(G,H)**, brown], SALL4 [**(I,J)**, brown], and CD44 [**(K,L)**, brown] in subcutaneous venous malformation (SCVM) **(A,C,E,G,I,K)** and intramuscular venous malformation (IMVM) **(B,D,F,H,J,L)**. Endothelial staining of all six embryonic stem cell markers was present on the endothelium within both SCVM and IMVM samples. Nanog **(A,B)**, pSTAT3 **(C,D)**, SOX2 **(G,H)**, and CD44 **(K,L)** were also expressed on cells (*arrowheads*) away from the endothelium in both SCVM and IMVM samples. Nuclei were counterstained with hematoxylin (blue). Original magnification: 400×.

Nanog (Figures 2A,B, red), pSTAT3 (Figures 2C,D, brown), OCT4 (Figures 2E,F, brown), SOX2 (Figures 2G,H, brown), SALL4 (Figures 2I,J, brown), and CD44 (Figures 2K,L, brown) were expressed on the endothelium of all seven samples of SCVM (Figures 2A,C,E,G,I,K) and seven samples of IMVM (Figures 2B,D,F,H,J,L). Interestingly, cells away from the endothelium also expressed Nanog (Figures 2A,B, red, *arrowheads*), pSTAT3 (Figures 2C,D, brown, *arrowheads*), SOX2 (Figures 2G,H, brown, *arrowheads*), and CD44 (Figures 2K,L, brown, *arrowheads*) in all samples of SCVM (Figures 2A,C,G,K) and IMVM (Figures 2B,D,H,L).

Positive staining was demonstrated in seminoma for Nanog (Image 1A in Supplementary Material, red), OCT4 (Image 1B in Supplementary Material, brown), and SALL4 (Image 1C in Supplementary Material, brown); skin for SOX2 (Image 1D in Supplementary Material, brown); and tonsil for pSTAT3 (Image S1E in Supplementary Material, brown) and CD44 (Image 1F in Supplementary Material, brown). Negative control SCVM (Image S1G in Supplementary Material, brown) and IMVM (Image 1H in Supplementary Material, brown) tissue samples demonstrated minimal staining.

### IF IHC Staining

**Figure 3:**
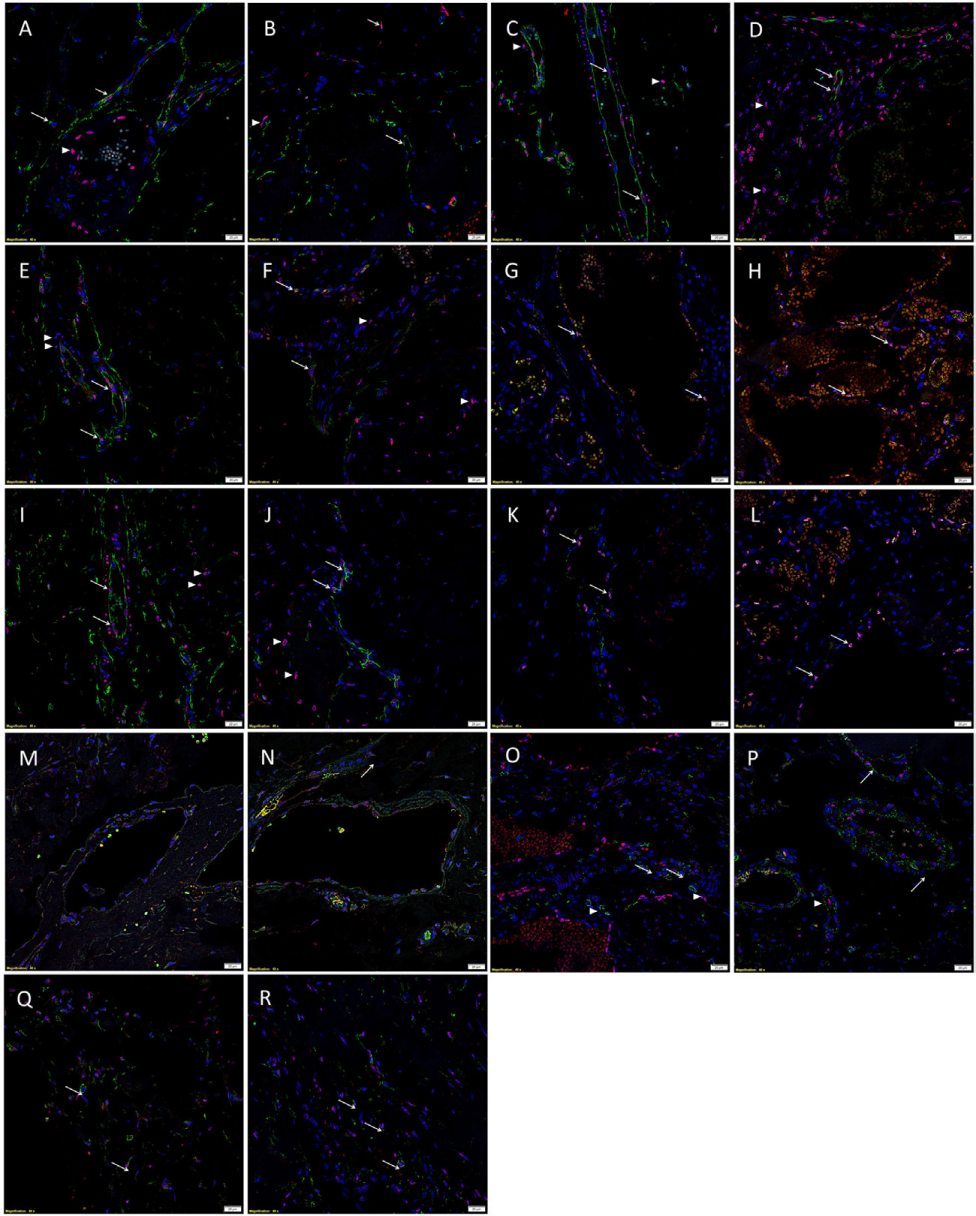
Representative immunofluorescent immunohistochemical-stained sections of subcutaneous venous malformation (SCVM) **(A)** and intramuscular venous malformation (IMVM) **(B)** samples, demonstrating the endothelium consisted of CD34^+^ (green)/ERG^−^ (red) (*long arrows*), ERG^+^(red)/CD34^−^ (green) endothelium (*arrowheads*), and CD34^+^ (red)/ERG^+^ (red) (*short arrows*) phenotypes. The CD34^+^ (green) endothelium expressed Nanog (red, *arrows*) in SCVM **(C)** and IMVM **(D)** lesions with cells away from the endothelium also expressing Nanog (red, *arrowheads*) within SCVM **(C)** and IMVM **(D)** lesions. The CD34^+^ (green) endothelium expressed pSTAT3 (red, *arrows*) in both SCVM **(E)** and IMVM **(F)** lesions. Cells away from the endothelium also expressed pSTAT3 (red, *arrowheads*) within SCVM **(E)** and IMVM **(F)** lesions. The ERG^+^ (red) endothelium also expressed OCT4 (green, *arrows*) in both SCVM **(G)** and IMVM **(H)** lesions. The CD34^+^ (green) endothelium expressed SOX2 (red, *arrows*) in SCVM **(I)** and IMVM **(J)** lesions. Cells away from the endothelium also expressed SOX2 (red, *arrowheads*) in SCVM **(I)** and IMVM **(J)** lesions. The ERG^+^ endothelium (red) expressed SALL4 (green, *arrows*) in SCVM **(K)** and IMVM **(L)** lesions. Dual staining of with SOX2 and SALL4 demonstrated the SALL4^+^ [**(M,N)**, green] endothelial population expressed SOX2 [**(M,N)**, red] in both SCVM **(M)** and IMVM **(N)**. The ERG^+^ endothelium (red) expressed CD44 (green, *arrows*) in SCVM **(O)** and IMVM **(P)** lesions with cells away from the endothelium also expressing CD44 (green, *arrowheads*) in SCVM **(O)** and IMVM **(P)**. Cells outside of the endothelium in both SCVM **(Q)** and IMVM **(R)** co-expressed Nanog [**(Q,R)**, red] and CD44 [**(Q,R)**, green]. Cell nuclei were counterstained with 4′,6′-diamidino-2-phenylindole [**(A–R)**, blue]. Scale bars: 20 µm.

Immunofluorescent IHC staining with CD34 (Figures 3A,B, green) and ERG (Figures 3A,B, red) demonstrated CD34^+^/ERG^−^ (*long arrows*), CD34^+^/ERG^+^ (*short arrows*), and CD34^−^/ERG^+^ (*arrowheads*) endothelium in SCVM (Figure 3A) and IMVM (Figure 3B) lesions. The CD34^+^ (Figures 3C,D, green) endothelium expressed Nanog in both SCVM (Figure 3C, red, *arrows*) and IMVM (Figure 3D, red, *arrows*) lesions with cells away from endothelium also expressing Nanog (Figures 3C,D, red, *arrowheads*) within SCVM (Figure 3C) and IMVM (Figure 3D) lesions. The CD34^+^ (Figures 3E,F, green) endothelium expressed pSTAT3 in both SCVM (Figure 3E, red) and IMVM (Figure 3F, red, *arrows*) lesions with cells away from the endothelium also expressing pSTAT3 (Figures 3E,F, red, *arrowheads*), within SCVM (Figure 3E) and IMVM (Figure 3F) lesions. The ERG^+^ (Figures 3G,H, red) endothelium expressed OCT4 in both the SCVM (Figure 3G, green, *arrows*) and IMVM (Figure 3H, green, *arrows*) lesions. The CD34^+^ (Figures 3I,J, green) endothelium expressed SOX2 in SCVM (Figure 3I, red, *arrows*) and IMVM (Figure 3J, red, *arrows*) lesions with cells away from the endothelium also expressing SOX2 (Figures 3I,J, red, *arrowheads*) within SCVM (Figure 3I) and IMVM (Figure 3J) lesions. The ERG^+^ (Figures 3K,L, red) endothelium expressed SALL4 in SCVM (Figure 3K, green, *arrows*) and IMVM (Figure 3L, green, *arrows*) lesions with no expression of SALL4 on the cells outside of the endothelium. To further characterize the SALL4^+^ (Figures 3M,N, green) endothelial population, we performed dual staining with SOX2, which confirmed co-expression of SOX2 (Figures 3M,N, red) in both SCVM (Figure 3M) and IMVM (Figure 3N). The ERG^+^ (Figures 3O,P, red) endothelium expressed CD44 in SCVM (Figure 3O, green, *arrows*) and IMVM (Figure 3P, green, *arrowheads*) lesions with cells away from the endothelium also expressing CD44 (Figures 3O,P, green, *arrowheads*) in SCVM (Figure 3O) and IMVM (Figure 3P) lesions. Dual IF IHC staining showed co-expression of Nanog (Figures 3Q,R, red, *arrows*) and CD44 (Figures 3Q,R, green, *arrows*) in cells outside of the endothelium within SCVM (Figure 3Q) and IMVM (Figure 3R) lesions, inferring the non-endothelial Nanog^+^ cells and the non-endothelial CD44^+^ cells are a single population. High powered images of the IF IHC-stained images in Figure 3 are presented in Image 2 in Supplementary Material.

Individual IF IHC staining for each of the aforementioned proteins shown in Figure 3 is presented in Images 3 and 4 in Supplementary Material for SCVM and IMVM, respectively. Negative controls for IF IHC staining for both SCVM and IMVM tissue samples demonstrated minimal staining (Image 5 in Supplementary Material).

### NanoString mRNA Analysis

**Figure 4:**
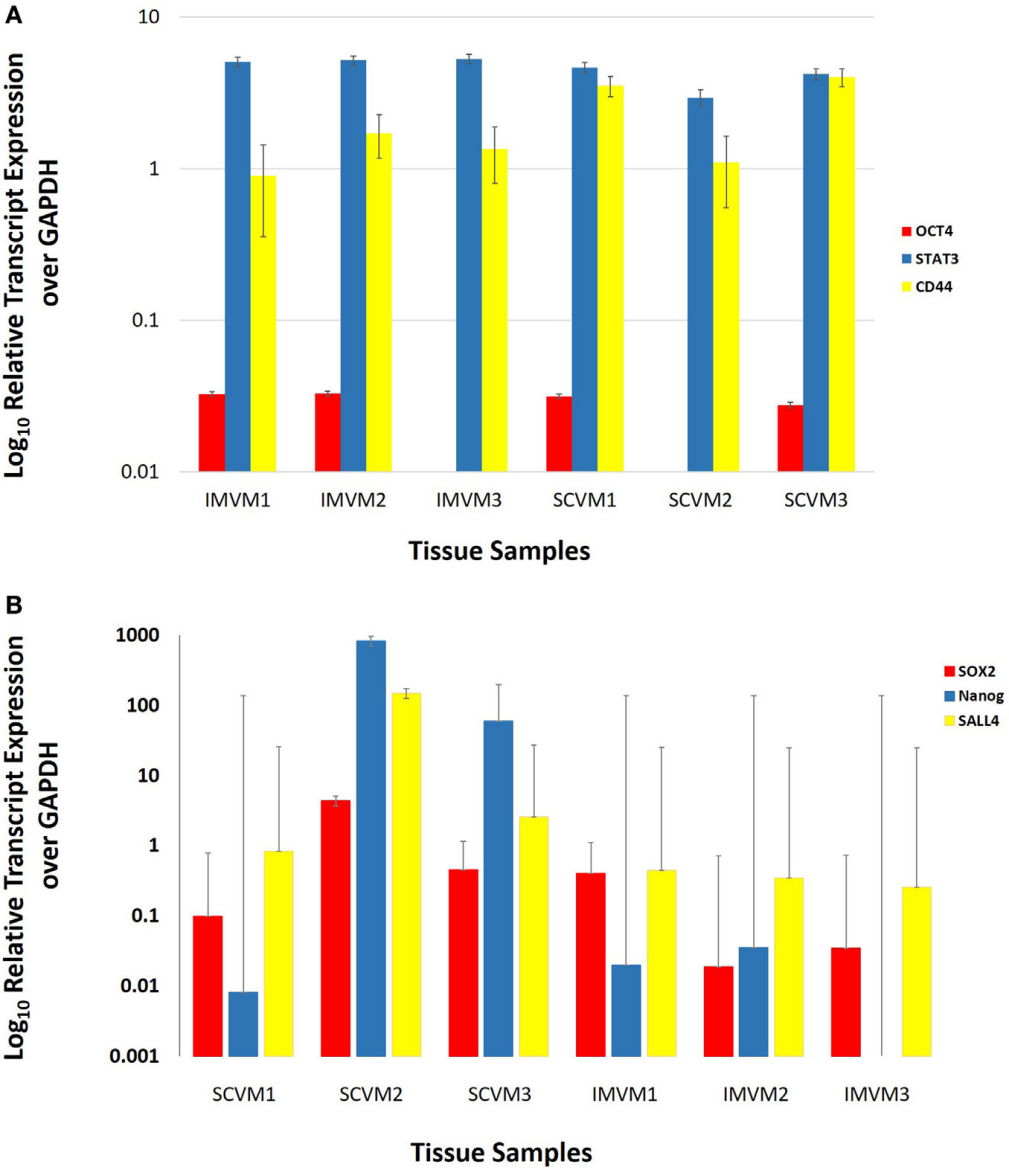
Log_10_ relative expression of OCT4, STAT3, and CD44 **(A)** and SOX2, Nanog, and SALL4 **(B)** mRNA transcripts in three subcutaneous venous malformation (SCVM) and three intramuscular venous malformation (IMVM) samples analyzed by NanoString **(A)** and RT-qPCR **(B)** analyses. Expression is depicted relative to the housekeeping gene GAPDH. OCT4 was detected in two SCVM and two IMVM samples **(A)**. STAT3 and CD44 **(A)** and SOX2 and SALL4 **(B)** were detected in all three samples. Nanog was detected in all three SCVM samples and two out of three IMVM samples **(B)**.

NanoString transcriptional profiling of three SCVM and three IMVM samples was normalized against the housekeeping gene GAPDH and averaged confirming the relative abundance of mRNA transcripts for STAT3 and CD44 in all SCVM and IMVM (Figure 4A). Statistical analysis of the mean values revealed no significant differences between the expression of STAT3 and CD44 between the SCVM and IMVM samples.

### RT-qPCR

Average expression levels of SOX2, SALL4, and Nanog genes, relative to the housekeeping gene GAPDH, are shown in Figure 4B. There were no significant differences between the mean expression levels of SOX2, SALL4, and Nanog between SCVM and IMVM samples.

## Discussion

98.8% of VMs arise sporadically. Familial VM that is typically multifocal is inherited in an autosomal dominant manner (2, 3, 5, 14, 19). The activating mutations of the tyrosine kinase receptor, TIE2, in the ECs account for the familial forms and 50% of sporadic (2, 3, 13, 14) VMs. The most common mutation in familial VM is R849W that involves an arginine-to-tryptophan substitution at position 849 in the kinase domain of TIE2 (2, 5, 14, 19). The most common somatic mutation is L914F, which accounts for 77% of patients with mutation-positive VM (14).

The exact mechanism by which mutant TIE2 leads to VMs is unknown (2). The mutations that lead to VM are located in the tyrosine kinase domain, kinase-insert domain, and carboxy terminal tail domains and cause ligand-independent receptor hyperphosphorylation *in vitro* and increased TIE2 activity (2, 5, 14, 19). The lack of correlation between phosphorylation and strength and severity of patient phenotype suggests a role in qualitative and not just quantitative anomalies in TIE2 signaling (2). The activating TIE2 mutation in ECs may reduce SMC ligand expression causing a local uncoupling between the normal recruitment of SMCs and the proliferation of ECs, resulting in affected vessels containing a disproportionately large number of ECs compared with SMCs (19).

Studies on mutant TIE2 have shown that expression of TIE2-L914F or TIE2-R849W in HUVECs increased activation of AKT and of STAT-1, an inflammatory mediator (2, 14). Elevated AKT signaling has an antiapoptotic effect on ECs leading to increased survival, as well as reducing the production of PDGF-B, which plays a major role in recruitment of mural cell (2, 14). Increased activity of this receptor tyrosine kinase that leading to abnormal sprouting and branching, which results in VMs, has been proposed (5). Vascular endothelial protein, tyrosine phosphatase, which is more strongly expressed in vessels invested with SMC than in capillaries and small veins, has been suggested to protect arteries and large veins from increased TIE2 activity, resulting in malformed venules/veins (5).

TIE2 is expressed on ECs, hematopoietic stem cells, and proangiogenic monocytes (14). Further research has identified ligands for the TIE2 receptor—Ang-1 and angiopoietin-2 (Ang-2) (5, 14). Ang-1 and Ang-2 bind to TIE2 and mediate, respectively, vascular maturation and angiogenesis (14). The TIE2 signaling pathway, through these proteins, is critical for EC–SMC communication in venous morphogenesis and is believed to play a role in regulating the assembly of non-endothelial components of the vessel including SMCs (3, 5). Knockout of TIE2 or Ang-1 in mice results in impaired blood vessel branching and deficient perivascular coverage (14). Deletion of Ang-1 in the developing embryo produces a disorganized vascular network with an increased number of ectatic vessels (14).

Involvement of activation of Tie2 receptor in the development of dilated luminal vessels derived from ESC (23). This led us to infer the putative presence of a primitive population within VM in the development of these ectatic vessels.

In this study, we have demonstrated expression of Nanog, pSTAT3, OCT4, SOX2, SALL4, and CD44 in the endothelium of both SCVM and IMVM. We have also demonstrated that Nanog, pSTAT3, SOX2, and CD44 are also expressed by cells outside of the endothelium, potentially by the same cells. These findings suggest the presence of at least two ESC-like subpopulations, one within and one outside of the endothelium of both SCVM and IMVM. Given that the endothelial ESC-like subpopulation expresses the more primitive marker OCT4 (27), it is exciting to speculate that they may give rise to the non-endothelial population. However, equally it is possible that there may be two distinct ESC-like subpopulations. Further confirmatory work on protein identification, using Western blotting, as well as *in vitro* and *in vivo* studies is needed to determine the precise relationship between these two ESC-like subpopulations. The functional role for each of these subpopulations and their contribution to VM pathology warrants further investigation although it is beyond the scope of this report. However, we believe that this is the first demonstration of the expression of these stem cell markers in VM endothelium and it would be interesting to investigate the endothelial expression of these markers in other vascular malformations.

This study describes an intriguing combined expression of ESC markers by the endothelium of both SCVM and IMVM lesions. Although some of these transcription factors, such as pSTAT3, may be associated with the normal hematopoiesis (28), we infer that its expression may be more related to its role in stem cell signaling (29). Furthermore, the expression of SOX2 and SALL4 are seen in both the cytoplasm and nucleus, which is supported by similar studies (30, 31), although the reasons for which are beyond the scope of this study. Interestingly, a recent report demonstrates the use of Y-27632, a Rho pathway inhibitor, for efficient culture of VM ECs (32), with the use of this cytokine previously reported to be crucial in the culture of ESCs (33).

The core nuclear transcription factors, such as Nanog, pSTAT3, and OCT4, have been used to identify and characterize the ESC population (34). OCT4 works synergistically with SOX2 and Nanog, to regulate various genes required for self-renewal and pluripotency (35, 36). The presence of leukemia inhibitory factor in pSTAT3 leads to its interaction with brachyury to form a loop stimulating the expression of Nanog (36).

Mogler et al. (22) show the presence of *c*-kit, a stem cell growth factor receptor, in the smaller, but not larger vessels of VM lesions. A potential explanation could be that the larger ectatic vessels are more mature and can no longer maintain a stem cell population, and the smaller vessels act as potential precursors.

Taken together, the novel findings in this report suggest a role of the ESC expression on the TIE2-activating mutation endothelium may possibly predispose to the formation VM phenotype, although this remains the topic of further investigation.

We have recently demonstrated the expression of PRR, a component of the RAS, on the endothelium of both SCVM and IMVM (20). PRR is known to signal through the Wnt/β-catenin pathway (37), and subsequently maintain pluripotency in ESCs (38). The finding of the two ESC-like subpopulations within SCVM and IMVM is novel and suggests that these primitive subpopulations may be a therapeutic target. Work is underway to investigate if these primitive subpopulations expressing the RAS, which can be manipulated by existing medications.

### Study Limitations

1. Larger studies are needed to confirm the significance of the findings in this study of a relatively small sample size.
2. *In vitro* and *in vivo* functional studies are needed to determine the ESC-like phenotype of these cells and relative role in the patho-etiology of VM.

## Ethics Statement

This study was carried out with the approval of the Central Health and Disability Ethics Committee (ref. no. 13/CEN/130) with written informed consent from all subjects in accordance with the Declaration of Helsinki.

## Author Contributions

TI and ST formulated the study hypothesis and designed the study. ET, SS, HB, TI, and ST analyzed the IHC data. TI analyzed the NanoString data. SE and ET performed and analyzed the qRT-PCR results. ET, SS, TI, and ST drafted the manuscript. All authors read and approved the manuscript.

## Conflict of Interest Statement

TI and ST are inventors of the patent application Treatment of Vascular Anomalies (62/287657), 2016. The authors otherwise declare that the research was conducted in the absence of any commercial or financial relationships that could be construed as a potential conflict of interest.

## Supplementary Material

Image 1 Positive controls for Nanog [**(A)**, red], OCT4 [**(B)**, brown], SALL4 [**(C)**, brown], SOX2 [**(D)**, brown], pSTAT3 [**(E)**, brown], CD44 [**(F)**, brown]. Negative controls for subcutaneous **(G)** and intramusacular **(H)** venous malformation. Nuclei were counter stained with hematoxylin (blue). Original magnification: **(A–F)** 400**×**; **(G,H)** 100**×**.

Image 2 High-powered images of immunofluorescent immunohistochemical-staining of SCVM **(A)** and IMVM **(B)** samples shown in Figure 3, demonstrating the endothelium consisted of CD34^+^ (green)/ERG^–^ (red), ERG^+^(red)/CD34^–^ (green) endothelium, and CD34+ (red)/ERG+ (red) phenotypes. The CD34+ (green) endothelium expressed Nanog (red) in SCVM **(C)** and IMVM **(D)** lesions with cells away from the endothelium also expressing Nanog (red) within SCVM **(C)** and IMVM **(D)** lesions. The CD34+ (green) endothelium expressed pSTAT3 (red) in both SCVM **(E)** and IMVM **(F)** lesions. Cells away from the endothelium also expressed pSTAT3 (red) within SCVM **(E)** and IMVM **(F)** lesions. The ERG+ (red) endothelium also expressed OCT4 (green) in both SCVM **(G)** and IMVM **(H)** lesions. The CD34+ (green) endothelium expressed SOX2 (red) in SCVM **(I)** and IMVM **(J)** lesions. Cells away from the endothelium also expressed SOX2 (red) in SCVM **(I)** and IMVM **(J)** lesions. The ERG+ endothelium (red) expressed SALL4 (green) in SCVM **(K)** and IMVM **(L)** lesions. Dual staining of with SOX2 and SALL4 demonstrated the SALL4+ [**(M,N)**, green] endothelial population expressed SOX2 [**(M,N)**, red] in both SCVM **(M)** and IMVM **(N)**. The ERG+ endothelium (red) expressed CD44 (green) in SCVM **(O)** and IMVM **(P)** lesions with cells away from the endothelium also expressing CD44 (green) in SCVM **(O)** and IMVM **(P)**. Cells outside of the endothelium in both SCVM **(Q)** and IMVM **(R)** co-expressed Nanog [**(Q,R)**, red] and CD44 [**(Q,R)**, green]. Cell nuclei were counterstained with 4**΄**,6**΄**-diamidino-2-phenylindole [**(A–R)**, blue]. Original magnification: 400**×**.

Image 3 Split immunofluorescent immunohistochemical stained images of subcutaneous venous malformation **(A,C,E,G,I,K,M,O,Q)** showing the expression of CD34 [**(A,C,E,I)**, green], ERG [**(B,H,L,P)**, red], Nanog [**(D,R)**, red], pSTAT3 [**(F)**, red], OCT4 [**(G)**, green], SOX2 [**(J,N)**, red], SALL4 [**(K,M)**, green], CD44 [**(O,Q)**, green]. Cell nuclei were counterstained with 4**΄**,6**΄**-diamidino-2-phenylindole [**(A–R)**, blue]. Scale bars: 20 μm.

Image 4 Split immunofluorescent immunohistochemical stained images of intramuscular venous malformation presented in Figure 3 (B,D,F,H,J,L,N,P,R) showing expression of CD34 [**(A,C,E,I)**, green], ERG [**(B,H,L,N)**, red], Nanog [**(D,R)**, red], pSTAT3 [**(F)**, red], OCT4 [**(H)**, green], SOX2 [**(J,P)**, red], SALL4 [**(K,O)**, green], CD44 [**(M,Q)**, green]. Cell nuclei were counterstained with 4**΄**,6**΄**-diamidino-2-phenylindole [**(A–R)**, blue]. Scale bars: 20 μm.

Image 5 Negative control immunofluorescent immunohistochemical sections of subcutaneous **(A)** and intramusacular **(B)** venous malformation demonstrating minimal staining. Cell nuclei were counterstained with 4**΄**,6**΄**-diamidino-2-phenylindole [**(A–F)**, blue]. Scale bars: 20 μm.

## Abbreviations

ACE, angiotensin-converting enzyme; Ang-1, angiopoietin 1; Ang-2, angiopoietin-2; ATIIR1, angiotensin II receptor 1; ATIIR2, angiotensin II receptor 2; DAB, 3,3′-diaminobenzidine; EC, endothelial cell; ESC, embryonic stem cell; H&E, hematoxylin and eosin; IF, immunofluorescent; IHC, immunohistochemical; IMVM, intramuscular venous malformation; PRR, prorenin receptor; RAS, renin–angiotensin system; SCVM, subcutaneous venous malformation; SMC, smooth muscle cell; VM, venous malformation.
