# Supplementary figures and images for: Embryonic Stem Cell-Like Subpopulations in Venous Malformation
Source: Front Med (Lausanne). 2017 Oct 4;4:162. doi: 10.3389/fmed.2017.00162 (PMC5632722; doi:10.3389/fmed.2017.00162)

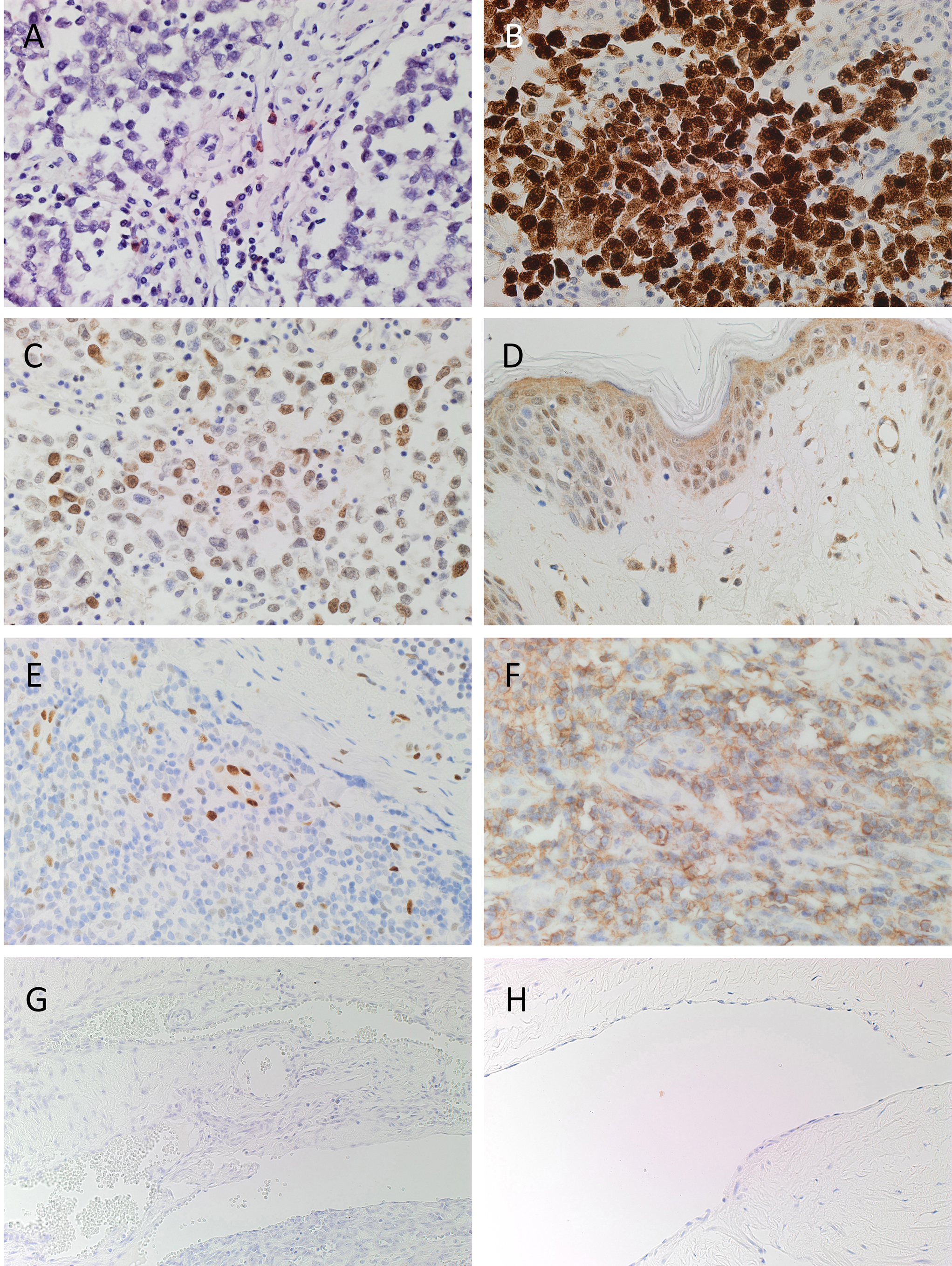

Supplement: Image 1 — Positive controls for Nanog [(A), red], OCT4 [(B), brown], SALL4 [(C), brown], SOX2 [(D), brown], pSTAT3 [(E), brown], CD44 [(F), brown]. Negative controls for subcutaneous (G) and intramusacular (H) venous malformation. Nuclei were counter stained with hematoxylin (blue). Original magnification: (A–F) 400×; (G,H) 100×. [file image_1.jpg]

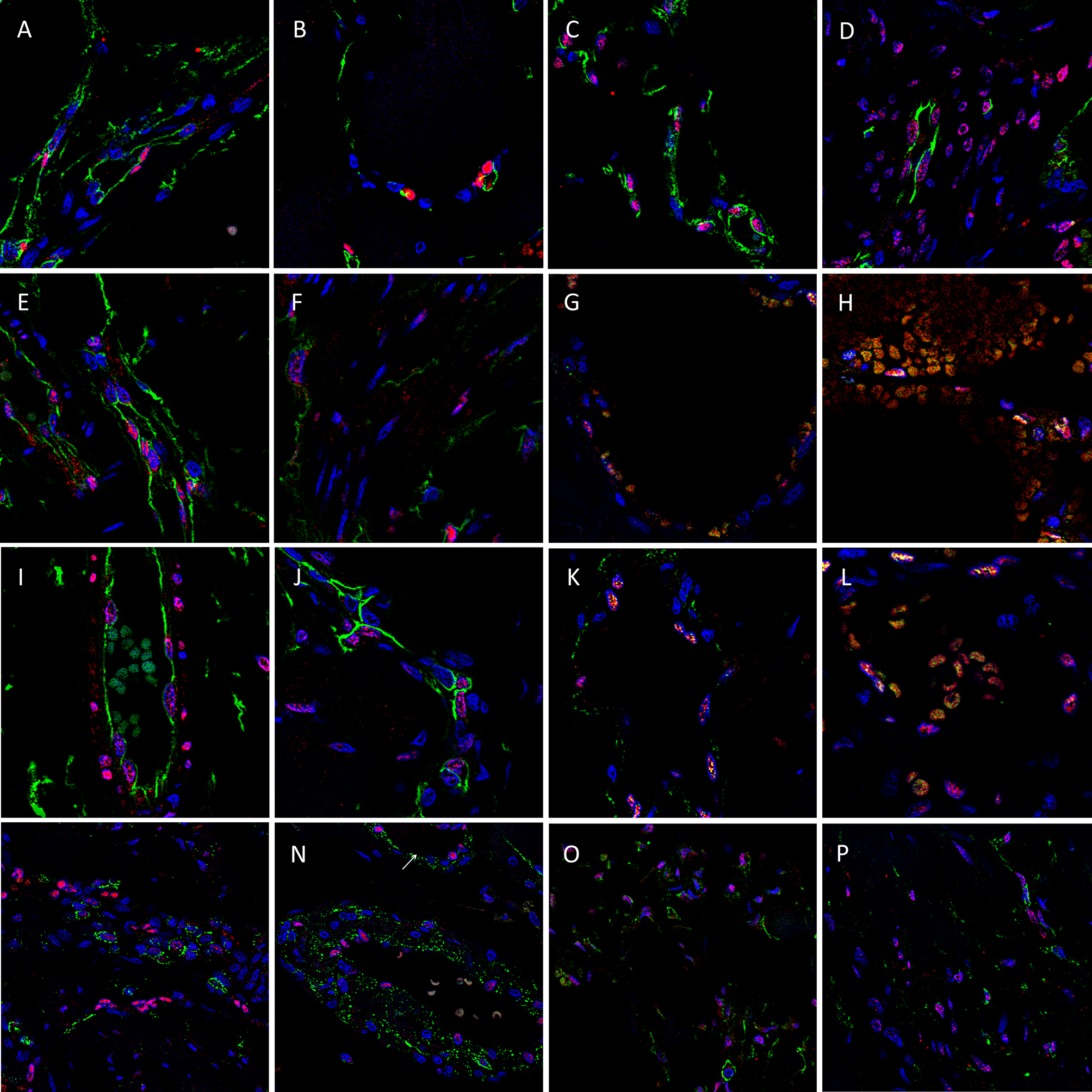

Supplement: Image 2 — High-powered images of immunofluorescent immunohistochemical-staining of SCVM (A) and IMVM (B) samples shown in Figure 3, demonstrating the endothelium consisted of CD34+ (green)/ERG– (red), ERG+(red)/CD34– (green) endothelium, and CD34+ (red)/ERG+ (red) phenotypes. The CD34+ (green) endothelium expressed Nanog (red) in SCVM (C) and IMVM (D) lesions with cells away from the endothelium also expressing Nanog (red) within SCVM (C) and IMVM (D) lesions. The CD34+ (green) endothelium expressed pSTAT3 (red) in both SCVM (E) and IMVM (F) lesions. Cells away from the endothelium also expressed pSTAT3 (red) within SCVM (E) and IMVM (F) lesions. The ERG+ (red) endothelium also expressed OCT4 (green) in both SCVM (G) and IMVM (H) lesions. The CD34+ (green) endothelium expressed SOX2 (red) in SCVM (I) and IMVM (J) lesions. Cells away from the endothelium also expressed SOX2 (red) in SCVM (I) and IMVM (J) lesions. The ERG+ endothelium (red) expressed SALL4 (green) in SCVM (K) and IMVM (L) lesions. Dual staining of with SOX2 and SALL4 demonstrated the SALL4+ [(M,N), green] endothelial population expressed SOX2 [(M,N), red] in both SCVM (M) and IMVM (N). The ERG+ endothelium (red) expressed CD44 (green) in SCVM (O) and IMVM (P) lesions with cells away from the endothelium also expressing CD44 (green) in SCVM (O) and IMVM (P). Cells outside of the endothelium in both SCVM (Q) and IMVM (R) co-expressed Nanog [(Q,R), red] and CD44 [(Q,R), green]. Cell nuclei were counterstained with 4΄,6΄-diamidino-2-phenylindole [(A–R), blue]. Original magnification: 400×. [file image_2.tif]

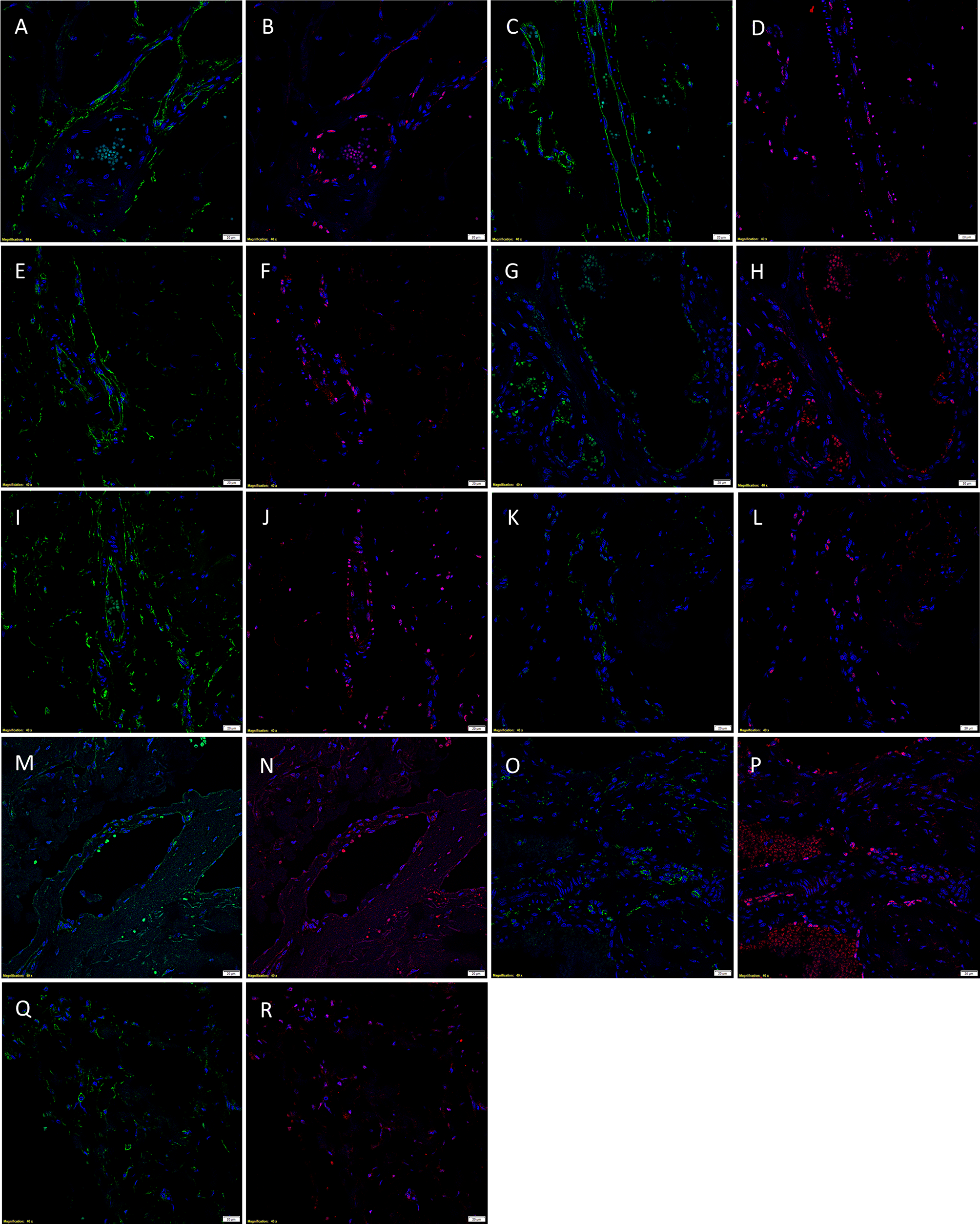

Supplement: Image 3 — Split immunofluorescent immunohistochemical stained images of subcutaneous venous malformation (A,C,E,G,I,K,M,O,Q) showing the expression of CD34 [(A,C,E,I), green], ERG [(B,H,L,P), red], Nanog [(D,R), red], pSTAT3 [(F), red], OCT4 [(G), green], SOX2 [(J,N), red], SALL4 [(K,M), green], CD44 [(O,Q), green]. Cell nuclei were counterstained with 4΄,6΄-diamidino-2-phenylindole [(A–R), blue]. Scale bars: 20 μm. [file image_3.tif]

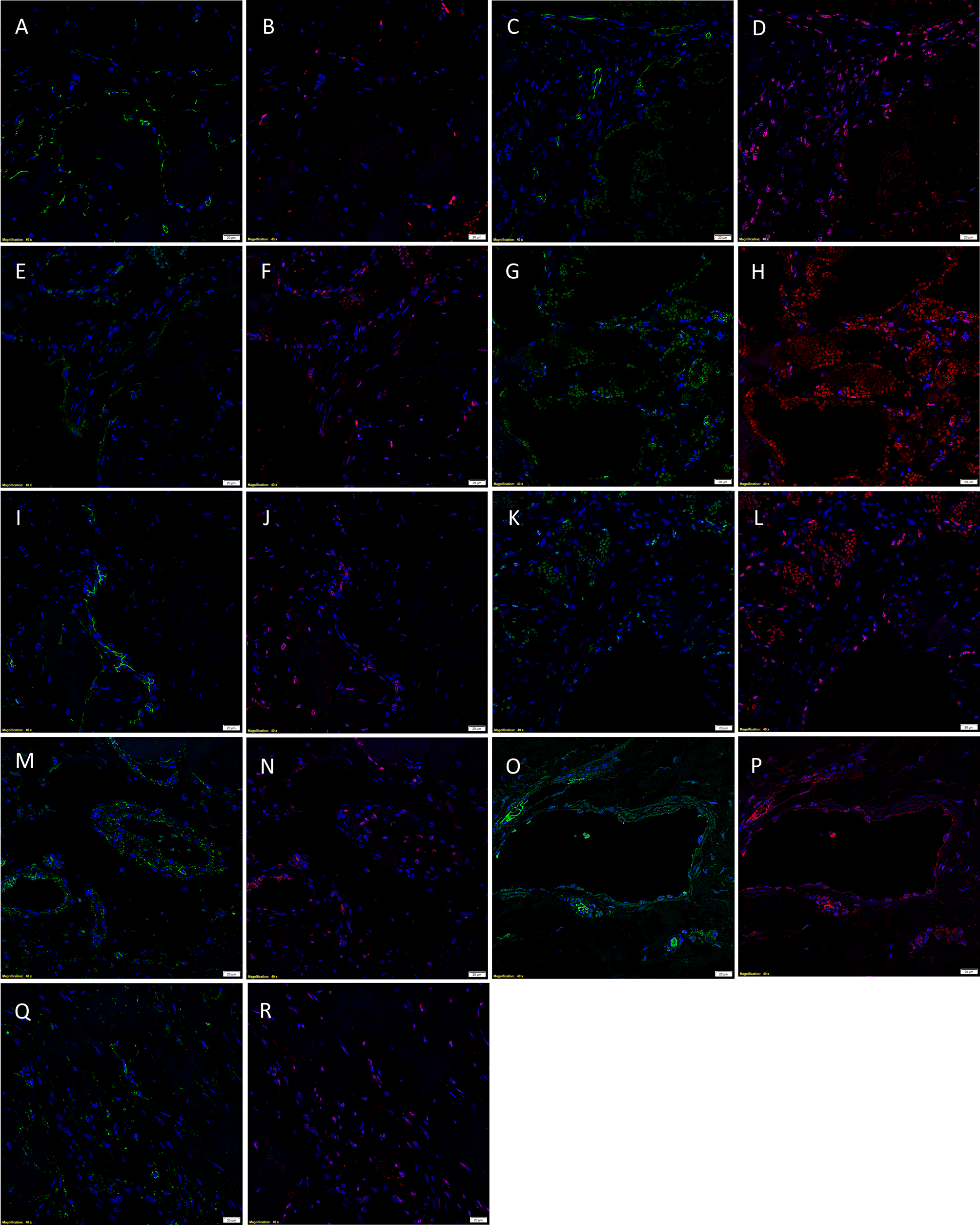

Supplement: Image 4 — Split immunofluorescent immunohistochemical stained images of intramuscular venous malformation presented in Figure 3 (B,D,F,H,J,L,N,P,R) showing expression of CD34 [(A,C,E,I), green], ERG [(B,H,L,N), red], Nanog [(D,R), red], pSTAT3 [(F), red], OCT4 [(H), green], SOX2 [(J,P), red], SALL4 [(K,O), green], CD44 [(M,Q), green]. Cell nuclei were counterstained with 4΄,6΄-diamidino-2-phenylindole [(A–R), blue]. Scale bars: 20 μm. [file image_4.tif]

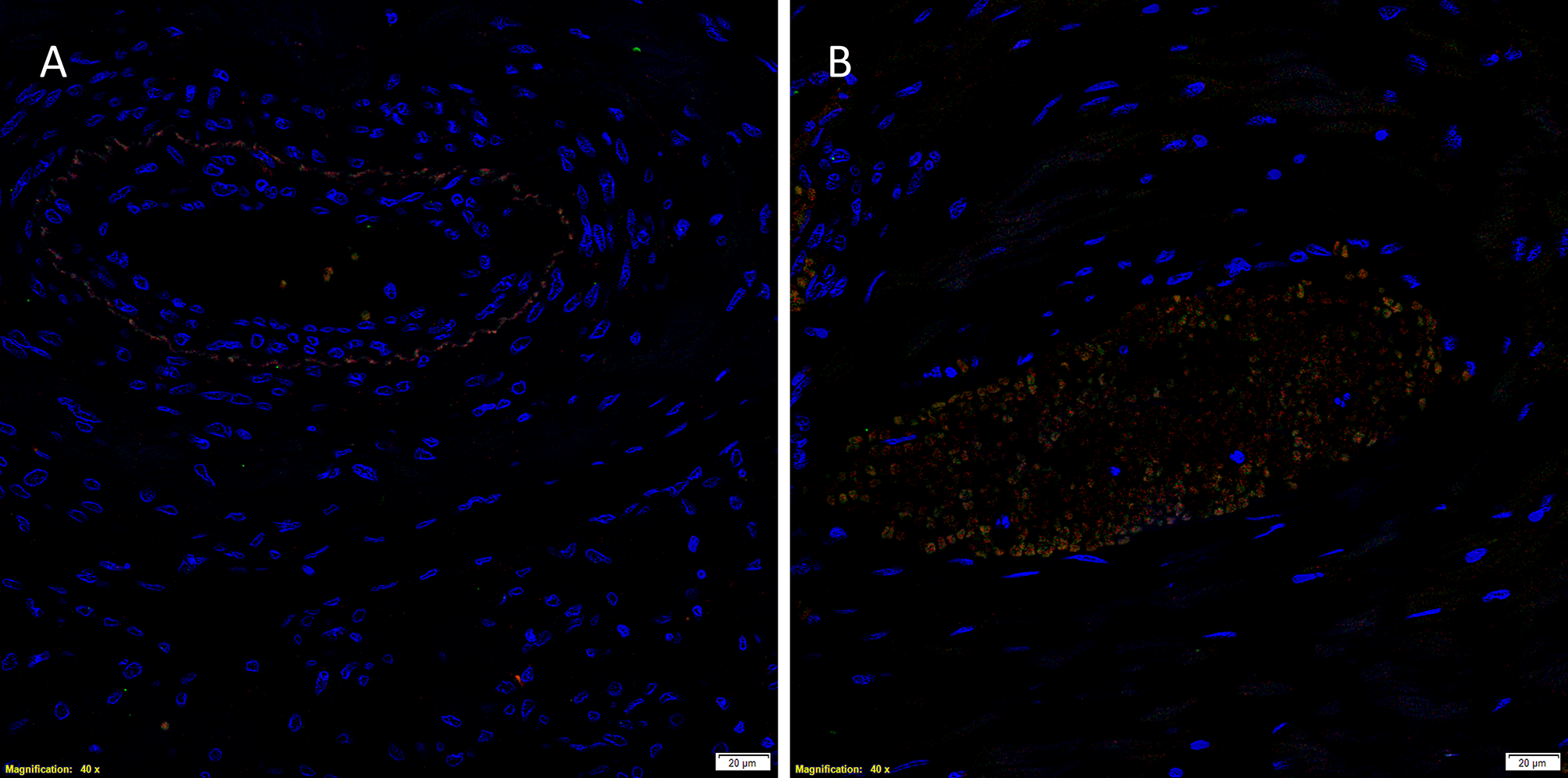

Supplement: Image 5 — Negative control immunofluorescent immunohistochemical sections of subcutaneous (A) and intramusacular (B) venous malformation demonstrating minimal staining. Cell nuclei were counterstained with 4΄,6΄-diamidino-2-phenylindole [(A–F), blue]. Scale bars: 20 μm. [file image_5.tif]
